# Supplementary material for: Global hypo-methylation in a proportion of glioblastoma enriched for an astrocytic signature is associated with increased invasion and altered immune landscape
Source: eLife. 2022 Nov 22;11:e77335. doi: 10.7554/eLife.77335 (PMC9681209; doi:10.7554/eLife.77335)
Supplement: Figure 2—source data 1. [file elife-77335-fig2-data1.zip › Figure_2_source_data_1/Figure_2I_J/homerResults/motif22.similar.html]

motif22

## Information for motif22

A
G
T
C
A
G
T
C
C
G
A
T
A
G
C
T
A
T
G
C
A
G
C
T
A
G
T
C
G
T
A
C
G
A
C
T
A
G
T
C
G
A
T
C
G
C
T
A
A
T
C
G
T
G
C
A
T
A
G
C
G
A
C
T
A
T
G
C
T
A
C
G
C
A
G
T
A
C
T
G
A
C
G
T
T
G
C
A
A
G
C
T
G
A
C
T
A
T
C
G
C
G
T
A
A
T
C
G
A
C
G
T
T
A
C
G
A
G
T
C
A
G
C
T
A
G
C
T
C
G
T
A
T
G
A
C
C
G
A
T
T
A
C
G
T
G
C
A
A
T
C
G
A
T
G
C
C
A
T
G
A
T
G
C
A
G
T
C
G
T
C
A
G
A
T
C
A
T
G
C
A
C
G
T
A
T
G
C
G
A
T
C
G
A
C
T
A
G
C
T
  
Reverse Opposite:  

T
C
G
A
C
T
G
A
C
T
A
G
T
A
C
G
T
G
C
A
T
A
C
G
C
T
A
G
C
A
G
T
T
C
A
G
T
A
C
G
G
T
A
C
T
A
C
G
T
A
G
C
A
C
G
T
A
T
G
C
G
C
T
A
A
C
T
G
G
C
A
T
T
C
G
A
T
C
G
A
T
C
A
G
A
T
G
C
T
G
C
A
T
A
G
C
C
G
A
T
T
A
G
C
C
T
G
A
T
C
G
A
A
C
G
T
T
G
C
A
T
G
A
C
G
T
C
A
A
T
G
C
T
A
C
G
C
T
G
A
A
T
C
G
A
C
G
T
T
A
G
C
C
G
A
T
C
A
T
G
C
T
A
G
C
T
G
A
C
A
T
G
C
T
A
G
T
C
G
A
T
A
C
G
T
C
G
A
G
C
T
A
T
C
A
G
T
C
A
G
  

|  |  |
| --- | --- |
| p-value: | 1e-33 |
| log p-value: | -7.762e+01 |
| Information Content per bp: | 1.430 |
| Number of Target Sequences with motif | 61.0 |
| Percentage of Target Sequences with motif | 8.40% |
| Number of Background Sequences with motif | 3.7 |
| Percentage of Background Sequences with motif | 1.29% |
| Average Position of motif in Targets | 95.0 +/- 45.4bp |
| Average Position of motif in Background | 53.2 +/- 7.5bp |
| Strand Bias (log2 ratio + to - strand density) | 0.3 |
| Multiplicity (# of sites on avg that occur together) | 1.08 |
| Motif File: | file (matrix) reverse opposite |

### Similar de novo motifs found

|  |  |  |  |  |  |  |  |
| --- | --- | --- | --- | --- | --- | --- | --- |
| Rank | Match Score | Redundant Motif | P-value | log P-value | % of Targets | % of Background | Motif file |
| 1 | 0.726 | A G T C C G T A C A T G C T G A C T A G A C G T T A G C T A C G C A G T A C T G A C G T G T C A A C G T C A G T A C T G G C A T A T C G G C A T T A C G G T A C G A C T C A G T T G C A G T A C C G A T T A C G T C G A A C T G T G A C T A C G G A T C G A T C G T C A G T A C G A T C G C A T A T G C G T A C G A C T A G C T | 1e-32 | -75.964993 | 5.51% | 0.52% | motif file (matrix) |
| 2 | 0.742 | A G C T G A T C T G A C C G T A C A T G C T G A C T A G A C G T A T G C T C A G C A G T A C T G A C G T G T C A A C G T A G C T C T A G G C T A T A C G C G A T A C T G G T A C A G C T A C G T T G C A G T A C C G A T A T C G C T G A A C T G T G A C C T A G G T A C A G T C G T C A A T G C G T A C G A C T A T G C G T A C G C A T A C G T A T C G A G C T A G T C | 1e-27 | -63.755690 | 6.34% | 1.03% | motif file (matrix) |
